# Unveiling the Relationships of Professional Identity, Values and Communication Competence in Nursing Students' Clinical Practice Behaviours: A Cross‐Sectional Study and Mediation Model Analysis

**DOI:** 10.1002/nop2.70270

**Published:** 2025-07-09

**Authors:** Li Yang, Si Ni Li, Qingyu Wang, Si Pan, Wen Zhao

**Affiliations:** ^1^ Clinical Nursing Teaching and Research Section, The Second Xiangya Hospital Central South University Changsha China; ^2^ The Nethersole School of Nursing, Faculty of Medicine The Chinese University of Hong Kong Sha Tin Hong Kong; ^3^ The Shenzhen Humanities & Social Sciences, Key Research Bases of the Center for Mental Health Shenzhen University Shenzhen China

**Keywords:** clinical communication competence, clinical practice behaviours, nursing education, nursing students, professional identity, professional values

## Abstract

**Aims:**

This study aimed to explore factors related to clinical practice behaviours and investigate the role of clinical communication competence in promoting professional identity/values and clinical practice behaviours among nursing students.

**Methods:**

Between July and August 2023, a cross‐sectional online survey were conducted among 791 nursing students undergoing clinical internships at a tertiary hospital in Hunan Province, China, using convenience sampling methods. The Professional Identity Questionnaire for Nurse Students, the Nurses Professional Values Scale‐Revised, the Clinical Communication Competence Scale and the Clinical Practice Behaviours Scale were used to assess students' professional identity, professional values, clinical communication competence and clinical practice behaviours, respectively. Descriptive statistics, *t*‐tests, ANOVA and Pearson's correlation were performed. Mediation models explored relationships between professional identity, professional values, communication competence and clinical practice behaviours, with communication competence as a mediator. Indirect effects were tested using bias‐corrected bootstrapping (5000 iterations) via the PROCESS macro.

**Results:**

The findings revealed that professional identity and professional values directly and indirectly influenced clinical practice behaviours in nursing students. Moreover, clinical communication competence was identified as a significant mediator in the relationship between professional identity (indirect effect = 0.11; 95% confidence interval [CI]: [0.09, 0.14], accounting for 23.4% of the total effect)/values (indirect effect = −0.13; 95% CI: [0.10, 0.17], accounting for 27.7% of the total effect) and clinical practice behaviours. Professional identity, professional values, clinical communication competence and clinical practice behaviours were found to have positive correlations with one another (Pearson's *r* ranged: 0.45–0.72, all *p*s < 0.05).

**Conclusion:**

This study sheds light on factors influencing nursing students' clinical practice behaviour, including professional identity, professional values and clinical communication competence. These insights can inform evidence‐based strategies for nursing education and practice, facilitating targeted interventions to enhance students' professional development and clinical competence.

**Patient or Public Contribution:**

No patient or public contribution was involved.

## Graphical abstract

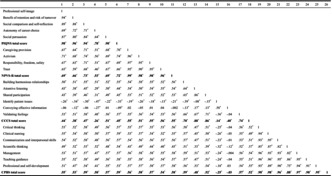

## 1 Introduction

The global nursing shortage has become a pressing issue in the field of healthcare, especially in the wake of major public health emergencies like the coronavirus disease (COVID‐19) pandemic. It is estimated that there is a global shortage of 5.9 million nurses (Buchan and Catton 2020). Over the next decade, one in six nurses is expected to retire, creating a demand for an additional 4.7 million nurses to fill these vacancies (Buchan and Catton 2020). In response, countries like the United States, United Kingdom and China have implemented policies to expand nursing college admissions, resulting in an increase in the number of licensed nurses (Wang et al. 2016; White 2017). Many nations are also improving nursing education and working conditions to address the shortage (Marć et al. 2019). However, while these interventions have effectively increased the quantity of nurses, high turnover rates among newly licensed nurses remain a significant challenge (Min et al. 2021). Previous studies have largely focused on addressing the supply–demand imbalance through workforce expansion and policy reforms, but they have paid insufficient attention to the qualitative aspects of preparing new nurses for the profession. Specifically, there is a lack of in‐depth exploration of how nursing education can address the underlying factors contributing to high turnover, such as the development of professional identity, the reinforcement of professional values and the cultivation of communication competence. These factors are crucial for equipping nursing students to handle the complexities of their professional roles and for promoting long‐term retention in the field.

Nursing education plays a key role in preparing students for their professional roles by fostering professional identity, instilling core values and developing essential skills (Brook et al. 2019; Hu et al. 2022; Yarbrough et al. 2017). This preparation goes beyond technical knowledge and clinical skills—it builds a sense of professional belonging, reinforces ethical principles and equips students to navigate the multifaceted challenges of clinical practice. By addressing these qualitative dimensions of nursing education, this study aims to fill the research gap and contribute to a deeper understanding of how professional identity, values and communication competence shape the development of nursing students and their clinical practice behaviours.

### 1.1 Professional Identity, Values, Communication Competence

Professional identity serves as a cornerstone for a nurse's professional development and is shaped throughout their educational journey. Nursing professional identity is defined as the values and beliefs that nurses possess, which guide their thoughts, actions and interactions with patients (Simmonds et al. 2020). It is a dynamic and continuous development process that starts before entering a pre‐registration programme and evolves throughout an individual's professional career (Johnson et al. 2012). It encompasses the beliefs, attitudes and values that individuals hold about their professional role and responsibilities. Developing a strong professional identity is vital for nursing students, as it helps them establish a sense of belonging, commitment and accountability to the nursing profession (Fitzgerald 2020). Previous studies have highlighted the importance of professional identity in professional values (Sun et al. 2016), career planning (Wei et al. 2021) and ethical decision‐making (Arries 2020), ultimately influencing their clinical practice behaviours (Kim 2017; Wei et al. 2021).

Professional values are principles and standards that guide nursing practice, reflecting the moral and ethical dimensions of the profession (Poorchangizi et al. 2019). The promotion of professional values in nursing starts when a student enters university and begins their nursing training, and it continues throughout their years of nursing practice (Bijani et al. 2019). These values, such as compassion, integrity and respect for autonomy, serve as a foundation for professional behaviour and decision‐making (Fahrenwald et al. 2005; Riklikiene et al. 2018). Nursing students who embrace these values are more likely to exhibit ethical clinical practice behaviours, including patient‐centred care, effective collaboration with healthcare teams and adherence to professional standards (Blais 2006). Importantly, these values also strongly predict high‐quality care, job satisfaction, motivation, organisational attachment and a strong sense of work commitment (Erkus and Dinc 2018).

Effective communication competence is crucial for nursing students to establish therapeutic relationships with patients, patients' families and interdisciplinary teams (Maureen Nokuthula 2018). Strong communication competence enables students to convey information accurately, listen attentively and respond empathetically (Bach and Grant 2015). Effective communication competence is essential for nurses as it fosters therapeutic relationships with patients, increases patient satisfaction, minimises treatment errors and ultimately improves the overall quality of nursing care and clinical practice through facilitating collaboration, patient education and the delivery of high‐quality care (Gutiérrez‐Puertas et al. 2020; Vaghee et al. 2018).

Overall, the development of professional identity, values and clinical communication competence is not only a fundamental aspect of nursing education but also plays a pivotal role in shaping the professional growth of nursing students. These factors have a profound influence on their clinical practice behaviours and overall competence as healthcare professionals. Clinical practice behaviours refer to the actions and conduct exhibited by healthcare professionals, such as nurses, in the clinical setting. These behaviours encompass a wide range of activities, including patient care, communication with patients and their families, collaboration with healthcare teams, adherence to professional standards and ethical guidelines, problem‐solving, critical thinking and decision‐making (Fukada 2018).

### 1.2 The Present Study

The clinical practice behaviours of nursing students play a crucial role in shaping their professional development as competent and compassionate healthcare providers. Previous empirical studies have consistently highlighted the relationships between professional identity, professional values and communication competence, as well as their influence on the clinical practice behaviours of nursing students. For example, a cross‐sectional study conducted in Korea with 140 nursing students from different universities found a significant correlation between professional identity and professional values (Min et al. 2021). Both variables were also significantly associated with clinical practice behaviours, such as problem‐solving skills (Min et al. 2021). Similarly, a cross‐sectional study in Turkey involving 262 clinical nurses demonstrated a moderate positive correlation between communication competence and care behaviours (Kirca and Bademli 2019). Despite the recognised importance of communication competence as an essential skill for nursing students, its specific role in relation to professional identity, professional values and clinical practice behaviours remains underexplored.

Therefore, this study aims to examine the mediating role of clinical communication competence in the relationship between professional identity, professional values and clinical practice behaviours. It seeks to explore the distinct contributions of each factor to the clinical practice behaviours of nursing students. By offering valuable insights into the determinants of these behaviours, the findings of this study are expected to inform the development of evidence‐based strategies that enhance nursing education and practice. Identifying areas for improvement will enable nursing educators and policymakers to design targeted interventions aimed at enhancing the clinical competence and professional development of nursing students.

## 2 Methods

### 2.1 Study Design

This study employed a cross‐sectional online survey design, following the guidelines outlined in the STrengthening the Reporting of Observational studies in Epidemiology (STROBE) statement (von Elm et al. 2007), to collect data.

### 2.2 Participants

Convenience sampling was used to recruit nursing students from various universities, colleges and schools who were undergoing clinical internships in a tertiary hospital in the Hunan province of mainland China between July and August 2023. This sampling method was selected for its practicality and efficiency in accessing a diverse group of participants within a limited timeframe, enabling timely data collection that reflects the real‐world clinical experiences of nursing students (Ahmed 2024).

An a priori calculation of the sample size was conducted based on the study by Sim et al. (2021), which indicated that at least 620 participants were required to construct a structural equation model with the smallest medium indirect effect size (0.4), four indicators and 0.4 loadings, using the bootstrap method. To account for potential non‐responses, an 11.1% non‐response rate was added, following a previous cross‐sectional study involving 544 Chinese nursing students (Zeng et al. 2019). Therefore, a minimum of 690 participants was determined to be necessary.

Participants were eligible for recruitment if they met the following criteria: (1) they were nursing students who had entered the graduation practice stage; (2) their internship duration exceeded 3 months and (3) they were capable of understanding and completing the questionnaires online via a mobile phone or computer.

### 2.3 Procedures

The Secretary of the Survey and Behavioural Research Ethics Committee of the Second Xiangya Hospital, Central South University, granted ethical approval for this study (Reference No. 20230207). Educators responsible for student management were provided with a link to an online questionnaire, which they subsequently shared with the nursing students. The purpose of the survey was clearly explained on the first page of the questionnaire, and respondents were required to provide a digital signature on a written informed consent form before proceeding.

The questionnaires were completed anonymously to ensure confidentiality, and the collected data will not be published in any public repository, except for use within this study. Respondents could only access the questionnaire after digitally signing the informed consent form. To further protect confidentiality, only the researcher had access to the data and all personal information (e.g., names) was encoded (e.g., NUR001) to safeguard participants' anonymity.

### 2.4 Measures

A general characteristics sheet was utilised to collect sociodemographic information from nursing students. This included data on various factors such as age, gender, marital status, ethnicity and religion. Additionally, the sheet gathered information on whether nursing was their first choice for the college entrance examination, the duration of their internship, the number of clinical practice departments they had experienced, their current satisfaction with the clinical practice environment, any unsatisfactory aspects of the clinical environment, their sense of achievement during the internship, their satisfaction with the nursing profession and their desired career path after graduation.

The *Professional Identity Questionnaire for Nurse Students* (*PIQNS*) is a 17‐item self‐report instrument measuring professional identity by rating on a 5‐point Likert scale (1—‘Very inconsistent’ to 5—‘Very consistent’) (Hao 2011). Higher scores on the PIQNS indicate a stronger professional identity among nursing students. This instrument was categorised into five subscales: professional self‐image, benefit of retention and risk of turnover, social comparison and self‐reflection, autonomy of career choice and social persuasion. The PIQNS has shown good internal consistency among Chinese nursing college students, with a Cronbach's *α* value of 0.83 for the total scale and ranging from 0.46 to 0.83 for the five subscales (Hao 2011). In our study, the Cronbach's *α* value for the PIQNS was 0.98, and the five subscales showed values ranging from 0.74 to 0.97.

The Chinese version of the *Nurses Professional Values Scale‐Revised* (*NPVS‐R*), translated and adapted from the original English version by Weis and Schank (2009), is a 26‐item self‐report questionnaire (Chen 2007). It assesses professional values by asking participants to rate each item on a 5‐point Likert scale, ranging from 1 (‘Not important’) to 5 (‘Most important’). Higher scores indicate a higher degree of professional values among nursing students. The NPVS‐R is categorised into four subscales of: (1) caregiving provision; (2) activism; (3) responsibility, freedom, safety and (4) trust. The Chinese version of the NPVS‐R has shown good internal consistency among Chinese nurses, with a Cronbach's *α* value of 0.76 for the total scale and ranging from 0.66 to 0.80 for the four domains (Chen 2007). This tool also exhibited acceptable 1‐week test–retest reliability among 45 nurses (*r* = 0.64). In our study, the Cronbach's *α* value for the NPVS‐R was 0.99, and the four subscales showed values ranging from 0.94 to 0.98.

The *Clinical Communication Competence Scale* (*CCCS*) is a self‐report instrument consisting of 28 items that measure clinical communication competence. Participants rate their competence on a 4‐point Likert scale, ranging from 1 (‘Never used’) to 4 (‘Frequently used’) (Yang et al. 2010). Higher scores indicate higher levels of clinical communication competence for nursing students. The scale includes six subscales: building harmonious relationships, attentive listening, shared participation, identify patient issues, conveying effective information and validating feelings. The CCCS has demonstrated good internal consistency among Chinese undergraduate and vocational college nursing students, with a Cronbach's *α* value of 0.84 for the total scale and ranging from 0.67 to 0.80 for the six subscales (Yang et al. 2010). In our study, the Cronbach's *α* value for the CCCS was 0.91, and the six subscales showed values ranging from 0.84 to 0.94.

The *Clinical Practice Behaviours Scale* (*CPBS*) is a 43 item self‐report instrument assessing clinical practice behaviours by rating on a 5‐point Likert scale (1—‘Very poor’ to 5—‘Very good’) (Jiang et al. 2006). Higher scores indicate higher levels of clinical practice behaviours for nursing students. The scale includes seven subscales: critical thinking, clinical nursing, communication and interpersonal skills, scientific thinking, management, teaching guidance, professional and self‐development. The CPBS has demonstrated good internal consistency among Chinese undergraduate nursing students, with a Cronbach's *α* value of 0.91 for the total scale and ranging from 0.60 to 0.82 for the seven subscales (Jiang et al. 2006). In our study, the Cronbach's *α* value for the CPBS was 0.93, and the seven subscales showed values ranging from 0.88 to 0.91.

### 2.5 Data Analysis

Descriptive statistics and correlational analyses were conducted using SPSS Statistical Software Version 25. Sociodemographic variables of nursing students were described using means, standard deviations and percentages. Differences in professional identity, professional values, clinical communication competence and clinical practice behaviours among nursing students were examined across various demographic variables, including gender, ethnicity, education level, major choice and career aspirations in the nursing field. Bivariate Pearson's correlation coefficients (*r*) were used to assess the associations between professional identity, professional values, communication competence and clinical practice behaviours. The effect sizes for the correlations were interpreted based on Cohen (2013)'s guidelines: > 0.10 indicating a small effect (i.e., the variable has a modest but noticeable influence on another variable), > 0.30 indicating a medium effect (i.e., the variable has a moderate impact on another variable) and > 0.50 indicating a large effect (i.e., a strong and substantial influence of the variable on another variable). Missing data at the item level were handled by prorating items if less than 25% of item‐level responses were missing. For entirely missing data, pairwise deletion was applied. The normality, linearity and homoscedasticity of all variables were assessed using Q–Q plots, skewness and kurtosis statistics and scatterplots. Differences in variables based on participants' general characteristics were analysed using independent *t*‐tests and analysis of variance (ANOVA) with Scheffé post hoc tests. A significance level of *p* < 0.05 was used to determine statistical significance for all analyses.

Two mediation models were constructed to investigate the relationships between professional identity (Model 1)/professional values (Model 2) and clinical practice behaviours, with communication competence as the mediating variable. Stepwise regression analyses were employed to explore the relationships between potential predictors. To test the mediating effects, the bias‐corrected bootstrapping method with 5000 iterations and bootstrapped 95% confidence intervals was applied using the PROCESS macro 4.1 (Model 4) developed by Hayes (2017). Mediation was considered significant when the confidence intervals of the indirect effects did not include zero. A variable was identified as a potential mediator if there was a significant relationship between the independent variable and the mediator, as well as between the mediator and the dependent variable. Notably, it was not necessary for the independent variable to have a direct significant relationship with the dependent variable, as mediation can still occur in the absence of this direct relationship (MacKinnon and Fairchild 2009).

## 3 Results

### 3.1 Characteristics of the Participants

Out of 880 completed questionnaires, 791 valid responses were obtained, yielding an 89.88% response rate after excluding missing or invalid data. The mean age of the students was 19.86 years (SD = 1.45), with 85.7% identifying as female and 100% being unmarried. A total of 80.8% of the students were from junior colleges, while 14.2% and 5.1% were from undergraduate programmes and vocational schools, respectively. Among the participants, 75.5% selected nursing as their first‐choice major in the college entrance examination, whereas 24.5% did not. Additionally, 67.0% expressed a willingness to pursue a career in the nursing profession, while 33.3% indicated they would not. The baseline sociodemographic characteristics of the participants are summarised in Table 1.

**TABLE 1:** Socio‐demographic characteristics of participants (*N* = 791).

Characteristics	*n* (%)
Age (mean, SD)	19.85 (1.45)
Gender	
Male	113 (14.3)
Female	678 (85.7)
Marital status	
Unmarried	791 (100)
Married	0 (0)
Ethnicity	
Han ethnicity	698 (88.2)
Ethnic minorities	93 (11.8)
Education level	
Vocational school	40 (5.1)
Junior college	639 (80.8)
Undergraduate	112 (14.2)
Nursing is a first‐choice major for the college/school entrance examination	
Yes	597 (75.5)
No	194 (24.5)
I am willing to pursue a career in the nursing field in the future	
Yes	530 (67.0)
No	261 (33.0)

### 3.2 Descriptive Statistics and Correlations Analyses

The skewness and kurtosis scores of the PIQNS, NPVS‐R, CCCS and CPBS fell within the acceptable range of −1.29 to 1.38, indicating that these variables meet the assumption of normality (see Table A1). The descriptive statistics for the PIQNS, NPVS‐R, CCCS and CPBS are also presented in Table A1. The mean score of PIQNS total score was 68.15 (SD: 12.95), with five subscales scores ranging from 7.62 (SD: 1.63) to 24.20 (SD: 4.94). The mean score of NPVS‐R total score was 106.97 (SD: 18.93), and the four subscales showed values ranging from 12.62 (SD: 2.27) to 41.40 (SD: 7.38). The mean score of CCCS total score was 88.72 (SD: 9.19), and the six subscales showed values ranging from 8.09 (SD: 1.71) to 19.22 (SD: 2.90). The mean score of CPBS total score was 176.43 (SD: 25.84), and the seven subscales showed values ranging from 15.51 (SD: 2.87) to 36.95 (SD: 5.44).

Tables A2, A3, A4, A5 provide an overview of disparities in professional identity, professional values, clinical communication competence and clinical practice behaviours among nursing students across various demographic variables. Students who prioritised nursing as their first‐choice major demonstrated significantly higher scores in professional identity, professional values, clinical communication competence and clinical practice behaviours compared to those who did not choose nursing as their first‐choice major. Similarly, students who expressed a desire to pursue a nursing career in the future exhibited significantly higher scores in these domains compared to those who did not intend to enter the field. Notably, there were significant differences in professional identity among students from different types of schools. Junior college nursing students displayed significantly higher levels of professional recognition compared to students from vocational schools and undergraduate nursing programmes. Furthermore, female nursing students demonstrated significantly higher clinical communication competence compared to their male counterparts.

Furthermore, Pearson's correlation analyses revealed significant associations between all variables and their respective subscales (refer to Table 2). Specifically, professional identity and professional values were positively associated with clinical communication competence and clinical practice behaviours, with effect sizes ranging from medium to large (ranging from 0.45 to 0.74).

**TABLE 2:** Correlation matrix.

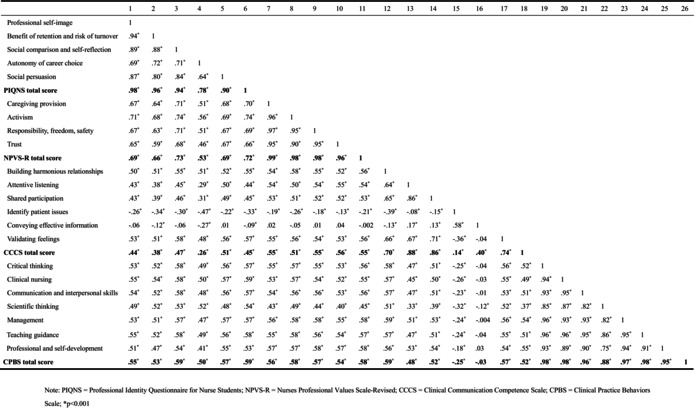

Abbreviations: CCCS, Clinical Communication Competence Scale; CPBS, Clinical Practice Behaviours Scale; NPVS‐*R*, Nurses Professional Values Scale‐Revised; PIQNS, Professional Identity Questionnaire for Nurse Students.

* *p* < 0.001.

### 3.3 Mediation Analyses

Two parallel mediation model analyses showed excellent model fit, with the Comparative Fit Index (CFI) and Tucker‐Lewis Index (TLI) both greater than 0.95, and the standardised root mean square residual (SRMR) and root mean square error of approximation (RMSEA) both smaller than 0.08. The results of the multiple stepwise regression analysis are presented in Table 3. Professional identity (*β* = 0.59, *t* = 20.48, *p* < 0.001) and professional values (*β* = 0.57, *t* = 19.58, *p* < 0.001) were found to significantly and positively predict clinical practice behaviours, with clinical communication competence serving as a mediating variable. These findings suggest that clinical communication competence partially mediates the relationship between the predictors (professional identity and professional values) and clinical practice behaviours.

**TABLE 3:** Results of stepwise regression analyses.

Regression model		Goodness of fit			Regression coefficient		
Outcome variables	Predictor variables	*R*	*R* ^2^	*F*	*β*	SE	*t*
Professional identity → clinical practice behaviours							
Clinical practice behaviours		0.59	0.35	419.60*			
	Professional identity				0.59	0.02	20.48*
Clinical communication competence		0.42	0.17	166.50*			
	Professional identity				0.42	0.01	12.90*
Clinical practice behaviours		0.66	0.44	305.00*			
	Professional identity				0.45	0.02	15.36*
	Clinical communication competence				0.33	0.05	11.16*
Professional values → clinical practice behaviours							
Clinical practice behaviours		0.57	0.33	383.41*			
	Professional identity				0.57	0.02	19.58*
Clinical communication competence		0.55	0.31	348.25*			
	Professional identity				0.55	0.01	18.66*
Clinical practice behaviours		0.62	0.39	246.83*			
	Professional identity				0.41	0.03	12.28*
	Clinical communication competence				0.29	0.06	8.63*

* *p* < 0.001.

The mediation effects of professional identity and professional values on clinical practice behaviours through clinical communication competence were further examined (see Table 4). The study revealed a significant total mediation effect for the relationship between professional identity and clinical practice behaviours (total effect = 0.47, SE = 0.02, 95% CI: [0.43, 0.52]), with clinical communication competence partially mediating this relationship (indirect effect = 0.11; SE = 0.01, 95% CI: [0.09, 0.14]), accounting for 23.4% of the total effect. Similarly, a significant total mediation effect was observed for the relationship between professional values and clinical practice behaviours (total effect = 0.47, SE = 0.02, 95% CI: [0.43, 0.52]). Clinical communication competence partially mediated this relationship as well (indirect effect = 0.13; SE = 0.02, 95% CI: [0.10, 0.17]), accounting for 27.7% of the total effect.

**TABLE 4:** Results of parallel mediation model analysis.

Model	Point estimate	BootSE	Boot 95% CI		Proportion mediated
			Lower	Upper	
Professional identity → Clinical communication competence → Clinical practice behaviours					
Total effects	0.47	0.02	0.43	0.52	—
Direct effects	0.36	0.02	0.32	0.41	76.6%
Indirect effects	0.11	0.01	0.09	0.14	23.4%
Professional values → Clinical communication competence → Clinical practice behaviours					
Total effects	0.47	0.02	0.43	0.52	
Direct effects	0.34	0.03	0.29	0.40	72.3%
Indirect effects	0.13	0.02	0.10	0.17	27.7%

## 4 Discussion

The present study examined the role of clinical communication competence in the relationships between professional identity/values and clinical practice behaviours among Chinese nursing students. The findings supported the mediating role of clinical communication competence in these relationships. Additionally, professional identity/values, clinical communication competence and clinical practice behaviours were found to be positively associated with one another.

The results demonstrated that professional identity and professional values exert both direct and indirect effects on clinical practice behaviours in nursing students. This finding is consistent with a previous scoping review that highlighted the critical role of professional identity and values in shaping nurses' behaviours, decision‐making processes and responses to political, social and healthcare reforms and advancements (Cornett et al. 2023). Developing a strong professional identity and adhering to professional values are essential for nursing students to foster a sense of commitment, accountability and ethical decision‐making in their clinical practice (Poorchangizi et al. 2019). Therefore, future nursing education should prioritise the cultivation of a strong professional identity and values among students. This can be achieved through a comprehensive curriculum that emphasises the importance of professionalism, ethical decision‐making and the core values underpinning nursing practice. Strategies such as mentorship programmes, reflective exercises and opportunities for self‐assessment can further support students in developing a robust professional identity and aligning their values with the nursing profession (Lutz et al. 2017).

The study identified clinical communication competence as a significant mediator in the relationship between professional identity/values and clinical practice behaviours. These findings, consistent with a previous cross‐sectional study involving 312 nursing professionals and 1369 nursing students, underscore the importance of communication competence—including social skills and emotional intelligence—in establishing therapeutic relationships, enhancing patient satisfaction and improving the overall quality of nursing care (Sanchis‐Giménez et al. 2023). Specifically, aspects of communication such as building harmonious relationships, attentive listening, shared participation, identifying patient issues, conveying effective information and validating feelings may play a pivotal role in shaping professional identity and values by fostering trust and mutual respect in patient and team interactions. Future studies could explore how specific aspects of communication, such as building harmonious relationships, attentive listening, shared participation, identifying patient issues, conveying effective information and validating feelings, shape professional identity and values. Longitudinal or experimental designs could examine their impact on trust and mutual respect in clinical settings, while qualitative research could provide deeper insights into their role in patient care and team dynamics. Such studies would inform targeted interventions and enhance nursing education and professional development programmes. Given the significant mediating role of clinical communication competence, it is essential to prioritise the development of effective communication skills in nursing education programmes. Equipping nursing students with competencies for establishing therapeutic relationships with patients and interdisciplinary teams can lead to improved patient outcomes, increased job satisfaction and reduced turnover rates (Arnold and Boggs 2019). To achieve this, nursing curricula should include dedicated training and courses focusing on communication techniques, active listening, empathy and patient‐centred care. Simulated scenarios and role‐playing exercises should also be incorporated to provide students with practical experience in communication competence (Gallimore and Rotzenberg 2023).

Furthermore, positive associations were identified between professional identity/values, clinical communication competence and clinical practice behaviours among Chinese nursing students. This significant finding suggests that these factors are interrelated and mutually reinforcing, carrying important implications for nursing education and practice. Nursing students with a strong professional identity and well‐established professional values are more likely to prioritise the development of their communication competence (Fitzgerald and Clukey 2021), which, in turn, leads to improved clinical practice behaviours (Guraya et al. 2021). This interplay between professional identity/values, clinical communication competence and clinical practice behaviours underscores the complex and dynamic nature of nursing education and practice. It highlights the necessity of adopting a comprehensive approach that addresses these interconnected factors. Future nursing education should focus on holistic training strategies, including the enhancement of clinical communication competence, the cultivation of a robust professional identity and values, the integration of interprofessional education, the incorporation of experiential learning and the promotion of continuous professional development (Hinkley et al. 2023). Moreover, providing opportunities for advanced training, specialisation and ongoing education can enable nurses to stay current with the latest evidence‐based practices, enhance their clinical competence and adapt to the rapidly evolving healthcare landscape (Mlambo et al. 2021).

In addition, students who prioritise nursing as their first‐choice major demonstrate higher levels of professional identity, professional values, clinical communication competence and clinical practice behaviours compared to those who did not choose nursing as their first‐choice major. This finding is consistent with a previous cross‐sectional study conducted with 262 Korean nursing students, which identified significant predictors of professional identity, including motivation for selecting nursing, satisfaction with nursing majors, self‐esteem and emotional labour (Nam and Lee 2016). These results suggest that students with a genuine interest and passion for nursing are more likely to exhibit positive attributes and behaviours associated with the profession. This underscores the importance of admitting students who are genuinely motivated to pursue nursing as a career, as they are more likely to possess the qualities and skills necessary for success in the field. Moreover, students expressing a desire to pursue a nursing career in the future also exhibit higher levels of professional identity, professional values, clinical communication competence and clinical practice behaviours compared to those who do not intend to enter the field. This finding aligns with a previous cross‐sectional study involving 453 full‐time undergraduate students in China, which reported a significantly positive correlation between nursing students' career planning and their professional identity (Wei et al. 2021). These findings emphasise the critical role of career aspirations and goals in shaping students' attitudes and behaviours. Students with a clear vision and strong motivation to pursue a nursing career are more likely to actively engage in their education, cultivate their professional identity and acquire the skills and competencies necessary for nursing practice (Poorchangizi et al. 2019).

Interestingly, the study reveals significant differences in professional identity among students from different types of schools, with junior college nursing students exhibiting higher levels of professional recognition compared to students in vocational schools and undergraduate nursing programmes. This finding aligns with a previous cross‐sectional study conducted with 398 nursing interns in China, which reported that students with higher levels of education exhibited lower levels of professional identity (Zeng et al. 2022). These results suggest that the educational environment and curriculum may play a critical role in shaping the development of professional identity among nursing students. Junior college nursing programmes may offer a more comprehensive and immersive educational experience, which fosters a stronger sense of professional identity compared to other types of nursing programmes. Therefore, to address the observed differences in professional identity among students from various educational backgrounds, nursing education programmes should critically evaluate and enhance their curricula to promote a strong sense of professional identity (Fitzgerald and Clukey 2021). Strategies can include incorporating experiential learning opportunities, interprofessional education and exposure to diverse nursing roles and specialties. Additionally, creating a supportive and inclusive learning environment that encourages collaboration and mentorship can further contribute to the development of professional identity across different types of nursing programmes (Vabo et al. 2021).

This study has limitations that deserve acknowledgement. Firstly, the cross‐sectional design limits the ability to investigate causal relationships between variables. To establish causation, future studies should adopt a longitudinal design with long‐term follow‐up. A longer timeframe would enable the examination of causal relationships and allow for mediation analyses to explore how clinical communication competence mediates the targeted outcomes. Secondly, the study's narrow focus on a specific province in mainland China and reliance on convenience sampling limit the generalizability of the findings. Further research using random sampling is recommended to obtain larger sample sizes from diverse geographical locations to conduct cross‐cultural or multi‐regional validation. This will enhance generalizability and provide a more comprehensive understanding as well as greater applicability of the topic. Third, the reliance on self‐reported data introduces potential biases, such as social desirability bias, which may affect the accuracy of the findings. Although self‐reported measures offer valuable insights into participants' perceptions, they are inherently subjective. Future research should incorporate observational methods or objective performance assessments to enhance data robustness and reduce bias. Lastly, the study's limited representation of male nursing students (14.3%) deserves attention. Notably, our findings indicate that male nursing students exhibit lower clinical communication competence compared to their female counterparts. This disparity may stem from societal expectations, a lack of male role models in nursing and potential bias or discrimination (Yip et al. 2021). To address this issue, nursing education programmes should implement targeted interventions, such as specialised communication training and support programmes, while fostering inclusivity and diversity (Charania and Patel 2022). Further research is necessary to investigate the underlying factors contributing to this disparity and to develop effective strategies for promoting equitable communication competence among male and female nursing students.

## 5 Conclusion

This study highlights professional identity, values and clinical communication competence as significant predictors of clinical practice behaviours among nursing students, with communication competence serving as a mediating factor. These findings emphasise the importance of incorporating professional identity development and communication skill enhancement into nursing education intervention guided by theoretical frameworks (e.g., Benner's Novice to Expert Model) (Thomas and Kellgren 2017). Strategies such as workshops, mentorship programmes and simulation‐based training could foster these competencies. Further research across diverse contexts is required to validate these findings and inform interventions to advance nursing education and improve clinical outcomes.

## Ethics Statement

The study was approved by the Secretary of Survey and Behavioural Research Ethics Committee of the Second Xiangya Hospital, Central South University (Reference No. 20230207). All the participants were required to read and understand the information sheet, and sign a consent form before data collection.

## Conflicts of Interest

The authors declare no conflicts of interest.

## Data Availability

The data presented in this study are available on request from the corresponding author. The data are not publicly available due to ethical reasons.

## Appendix A

**TABLE A1:** Descriptive statistics and the skewness and kurtosis scores among variables (*N* = 791).

	Mean	SD	Skewness	Kurtosis
Professional Identity Questionnaire for Nurse Students				
Total score	68.15	12.95	−0.538	0.224
Professional self‐image	24.20	4.94	−0.640	0.249
Benefit of retention and risk of turnover	15.72	3.37	−0.520	0.051
Social comparison and self‐reflection	12.21	2.32	−0.514	0.067
Autonomy of career choice	7.62	1.63	−0.100	−0.331
Social persuasion	8.40	1.59	−0.905	0.759
Nurses Professional Values Scale‐Revised				
Total score	106.97	18.93	−0.553	−0.083
Caregiving provision	41.40	7.38	−0.621	0.093
Activism	32.26	5.93	−0.401	−0.283
Responsibility, freedom, safety	20.69	3.68	−0.529	−0.252
Trust	12.62	2.27	−0.697	−0.092
Clinical Communication Competence Scale				
Total score	88.72	9.19	−0.704	0.444
Building harmonious relationships	19.22	2.90	−0.219	0.275
Attentive listening	18.06	2.54	−1.285	1.380
Shared participation	14.29	2.09	−1.082	0.769
Identify patient issues	12.20	2.93	0.222	−0.552
Conveying effective information	8.09	1.71	0.504	−0.684
Validating feelings	16.85	2.79	−0.493	−0.310
Clinical Practice Behaviours Scale				
Total score	176.43	25.84	−0.156	0.052
Critical thinking	36.95	5.44	−0.146	0.063
Clinical nursing	24.53	3.85	−0.165	−0.211
Communication and interpersonal skills	16.47	2.52	−0.201	−0.110
Scientific thinking	15.51	2.87	−0.047	−0.499
Management	24.85	3.64	−0.260	0.134
Teaching guidance	32.82	4.91	−0.161	−0.026
Professional and self‐development	25.29	3.60	−0.429	0.334

**TABLE A2:** Differences in Professional Identity Questionnaire for Nurse Students across demographic variables (*N* = 791).

	Total score		Professional self‐image		Benefit of retention and risk of turnover		Social comparison and self‐reflection		Autonomy of career choice		Social persuasion	
	M ± SD	*t*/*F* (*p*)	M ± SD	*t*/*F* (*p*)	M ± SD	*t*/*F* (*p*)	M ± SD	*t*/*F* (*p*)	M ± SD	*t*/*F* (*p*)	M ± SD	*t*/*F* (*p*)
Gender												
Male	69.42 ± 14.22	1.12	24.88 ± 5.08	1.57	16.05 ± 3.49	1.15	12.27 ± 2.60	0.29	7.74 ± 1.80	0.84	8.47 ± 1.83	0.52
Female	67.94 ± 12.73	(0.264)	24.09 ± 4.91	(0.116)	15.66 ± 3.35	(0.253)	12.21 ± 2.27	(0.760)	7.60 ± 1.60	(0.403)	8.38 ± 1.55	(0.603)
Ethnicity												
Han ethnicity	68.05 ± 13.06	−0.63	24.16 ± 4.98	−0.70	15.69 ± 3.42	−0.63	12.20 ± 2.34	−0.62	7.61 ± 1.63	−0.81	8.40 ± 1.59	0.13
Ethnic minorities	68.95 ± 12.17	(0.531)	24.54 ± 4.59	(0.484)	15.92 ± 3.02	(0.527)	12.35 ± 2.15	(0.536)	7.75 ± 1.59	(0.420)	8.38 ± 1.58	(0.894)
Education level												
Vocational school	66.15 ± 12.01	**6.27**	23.53 ± 5.04	**6.60**	15.25 ± 3.17	**5.66**	11.68 ± 2.00	**4.39**	7.35 ± 1.49	2.98	8.35 ± 1.41	**7.56**
Junior college	68.93 ± 12.87	**(0.002)**	24.50 ± 4.86	**(0.001)**	15.91 ± 3.35	**(0.004)**	12.33 ± 2.33	**(0.013)**	7.69 ± 1.64	(0.052)	8.49 ± 1.58	**(0.001)**
Undergraduate	64.46 ± 13.15	**b > c**	22.73 ± 5.10	**b > c**	14.79 ± 3.46	**b > c**	11.73 ± 2.26	**b > c**	7.33 ± 1.61		7.87 ± 1.64	**b > c**
Nursing is a first‐choice major for the college/school entrance examination												
Yes	69.54 ± 12.30	**5.38**	24.78 ± 4.72	**5.93**	16.04 ± 3.22	**4.74**	12.43 ± 2.22	**4.65**	7.71 ± 1.59	2.66	8.58 ± 1.46	**5.84**
No	63.88 ± 13.98	**(** ***** **)**	22.41 ± 5.17	**(** ***** **)**	14.73 ± 3.64	**(** ***** **)**	11.55 ± 2.48	**(** ***** **)**	7.36 ± 1.72	(0.008)	7.83 ± 1.83	**(** ***** **)**
I am willing to pursue a career in the nursing field in the future												
Yes	72.75 ± 10.43	**16.47**	26.08 ± 3.81	**18.19**	16.86 ± 2.74	**15.54**	12.92 ± 1.99	**13.64**	7.96 ± 1.59	8.58	8.92 ± 1.27	**14.96**
No	58.82 ± 12.57	**(** ***** **)**	20.38 ± 4.75	**(** ***** **)**	13.39 ± 3.35	**(** ***** **)**	10.77 ± 2.27	**(** ***** **)**	6.95 ± 1.50	**(** * **)**	7.33 ± 1.65	**(** ***** **)**

*Note:* The bolded t‐values in the table indicate significant differences.

* *p* < 0.001.

**TABLE A3:** Differences in Nurses Professional Values Scale across demographic variables (*N* = 791).

	Total score		Caregiving provision		Activism		Responsibility, freedom, safety		Trust	
	M ± SD	*t*/*F* (*p*)	M ± SD	*t*/*F* (*p*)	M ± SD	*t*/*F* (*p*)	M ± SD	*t*/*F* (*p*)	M ± SD	*t*/*F* (*p*)
Gender										
Male	108.51 ± 20.38	0.93	41.87 ± 8.05	0.72	32.79 ± 6.46	1.03	21.05 ± 3.83	1.14	12.81 ± 2.36	0.92
Female	106.72 ± 18.68	(0.351)	41.33 ± 7.27	(0.471)	32.17 ± 5.84	(0.305)	20.63 ± 3.66	(0.257)	12.59 ± 2.26	(0.358)
Ethnicity										
Han ethnicity	107.02 ± 19.08	0.21	41.44 ± 7.44	0.40	32.26 ± 5.97	0.02	20.70 ± 3.72	0.21	12.62 ± 2.29	0.05
Ethnic minorities	106.59 ± 17.90	(0.836)	41.12 ± 6.95	(0.692)	32.25 ± 5.66	(0.985)	20.61 ± 3.46	(0.832)	12.61 ± 2.17	(0.963)
Education level										
Vocational school	102.40 ± 17.31	2.45	39.78 ± 6.76	2.19	30.80 ± 5.52	2.51	19.63 ± 3.39	2.31	12.20 ± 2.00	1.61
Junior college	107.67 ± 19.15	(0.087)	41.66 ± 7.48	(0.112)	32.51 ± 5.99	(0.065)	20.80 ± 3.73	(0.100)	12.69 ± 2.30	(0.201)
Undergraduate	104.65 ± 17.91		40.51 ± 6.94		31.35 ± 5.64		20.41 ± 3.50		12.38 ± 2.21	
Nursing is a first‐choice major for the college/school entrance examination										
Yes	108.04 ± 18.69	**2.79**	41.81 ± 7.28	**2.75**	32.58 ± 5.84	**2.66**	20.90 ± 3.68	**2.83**	12.75 ± 2.22	**2.81**
No	103.69 ± 19.34	**(0.005)**	40.14 ± 7.57	**(0.006)**	31.28 ± 6.12	**(0.008)**	20.04 ± 3.64	**(0.005)**	12.23 ± 2.38	**(0.005)**
I am willing to pursue a career in the nursing field in the future										
Yes	111.58 ± 16.98	**10.39**	43.14 ± 6.61	**10.02**	33.70 ± 5.40	**10.38**	21.57 ± 3.32	**10.13**	13.17 ± 2.00	**10.22**
No	97.62 ± 19.28	(*)	37.87 ± 7.62	**(** ***** **)**	29.33 ± 5.90	**(** ***** **)**	18.91 ± 3.75	**(** ***** **)**	11.52 ± 2.39	**(** ***** **)**

*Note:* The bolded t‐values in the table indicate significant differences.

* *p* < 0.001.

**TABLE A4:** Differences in Clinical Communication Competence Scale for Nurse Students across demographic variables (*N* = 791).

	Total score		Building harmonious relationships		Attentive listening		Shared participation		Identify patient issues		Conveying effective information		Validating feelings	
	M ± SD	*t*/*F* (*p*)	M ± SD	*t*/*F* (*p*)	M ± SD	*t*/*F* (*p*)	M ± SD	*t*/*F* (*p*)	M ± SD	*t*/*F* (*p*)	M ± SD	*t*/*F* (*p*)	M ± SD	*t*/*F* (*p*)
Gender														
Male	86.92 ± 9.50	**−2.26**	19.06 ± 3.29	−0.62	17.67 ± 2.80	−1.78	13.98 ± 2.35	−1.72	11.67 ± 2.68	**−2.09**	7.70 ± 1.60	**−2.66**	16.83 ± 3.01	−0.07
Female	89.02 ± 9.11	**(0.024)**	19.24 ± 2.83	(0.538)	18.13 ± 2.49	(0.076)	14.35 ± 2.03	(0.086)	12.29 ± 2.96	**(0.037)**	8.16 ± 1.72	**(0.008)**	16.85 ± 2.75	(0.946)
Ethnicity														
Han ethnicity	88.76 ± 9.22	0.32	19.20 ± 2.91	−0.37	18.08 ± 2.56	0.44	14.30 ± 2.10	0.02	12.25 ± 2.95	1.09	8.12 ± 1.73	1.01	16.82 ± 2.79	−0.76
Ethnic minorities	88.44 ± 8.99	(0.753)	19.32 ± 2.83	(0.710)	17.96 ± 2.38	(0.664)	14.29 ± 2.00	(0.983)	11.89 ± 2.73	(0.274)	7.92 ± 1.57	(0.311)	17.05 ± 2.75	(0.449)
Education level														
Vocational school	87.45 ± 9.82	1.29	19.00 ± 2.74	0.41	17.73 ± 2.59	1.51	13.95 ± 2.10	2,82	12.48 ± 3.15	**3.25**	8.03 ± 1.58	0.26	16.28 ± 2.67	**7.70**
Junior college	88.98 ± 9.09	(0.275)	19.26 ± 2.87	(0.663)	18.14 ± 2.52	(0.222)	14.38 ± 2.10	(0.060)	12.08 ± 2.94	**(0.039)**	8.08 ± 1.74	(0.775)	17.03 ± 2.77	**(** ***** **)**
Undergraduate	87.72 ± 9.49		19.04 ± 3.12		17.75 ± 2.61		13.93 ± 1.98		12.82 ± 2.71	**c > b**	8.20 ± 1.60		15.99 ± 2.77	**b > c**
Nursing is a first‐choice major for the college/school entrance examination														
Yes	89.54 ± 9.00	**4.44**	19.33 ± 2.84	1.92	18.28 ± 2.42	**4.20**	14.44 ± 2.02	**3.48**	12.34 ± 3.04	**2.31**	8.16 ± 1.75	1.85	16.99 ± 2.76	**2.58**
No	86.21 ± 9.33	**(** ***** **)**	18.87 ± 3.04	(0.055)	17.41 ± 2.78	**(** ***** **)**	13.85 ± 2.23	**(0.001)**	11.78 ± 2.53	**(0.021)**	7.90 ± 1.56	(0.065)	16.40 ± 2.84	**(0.010)**
I am willing to pursue a career in the nursing field in the future														
Yes	90.22 ± 8.46	**6.73**	19.56 ± 2.78	**4.78**	18.48 ± 2.27	**6.67**	14.59 ± 1.94	**5.73**	12.19 ± 3.10	−0.25	8.15 ± 1.80	1.39	17.26 ± 2.64	**6.12**
No	85.67 ± 9.86	**(** ***** **)**	18.52 ± 3.01	**(** ***** **)**	17.23 ± 2.84	**(** ***** **)**	13.70 ± 2.25	**(** ***** **)**	12.24 ± 2.56	(0.806)	7.97 ± 1.51	(0.165)	16.00 ± 2.89	**(** ***** **)**

*Note:* The bolded t‐values in the table indicate significant differences.

* *p* < 0.001.

**TABLE A5:** Differences in Clinical Practice Behaviours Scale for Nurse Students across demographic variables (*N* = 791).

	Total score		Critical thinking		Clinical nursing		Communication and interpersonal skills		Scientific thinking		Management		Teaching guidance		Professional and self‐development	
	M ± SD	*t*/*F* (*p*)	M ± SD	*t*/*F* (*p*)	M ± SD	*t*/*F* (*p*)	M ± SD	*t*/*F* (*p*)	M ± SD	*t*/*F* (*p*)	M ± SD	*t*/*F* (*p*)	M ± SD	*t*/*F* (*p*)	M ± SD	*t*/*F* (*p*)
Gender																
Male	180.04 ± 26.28	1.61	37.64 ± 5.63	1.46	25.07 ± 3.84	1.62	16.68 ± 2.59	0.96	16.03 ± 2.88	**2.06**	25.32 ± 3.74	1.47	33.53 ± 4.86	1.66	25.78 ± 3.78	1.55
Female	175.83 ± 25.74	(0.108)	36.83 ± 5.41	(0.145)	24.44 ± 3.85	(0.105)	16.44 ± 2.50	(0.338)	15.43 ± 2.86	**(0.040)**	24.78 ± 3.62	(0.143)	32.71 ± 4.91	(0.098)	25.21 ± 3.57	(0.122)
Ethnicity																
Han ethnicity	176.38 ± 25.74	−0.15	36.94 ± 5.42	−0.14	24.51 ± 3.84	−0.26	16.47 ± 2.51	0.08	15.48 ± 2.87	−0.81	24.86 ± 3.64	0.05	32.81 ± 4.91	−0.24	25.31 ± 3.57	0.28
Ethnic minorities	176.81 ± 26.73	(0.882)	37.02 ± 5.67	(0.888)	24.62 ± 3.95	(0.797)	16.45 ± 2.58	(0.935)	15.74 ± 2.84	(0.416)	24.84 ± 3.70	(0.964)	32.94 ± 4.92	(0.814)	25.19 ± 3.88	(0.776)
Education level																
Vocational school	169.53 ± 28.29	2.95	35.63 ± 6.02	2.92	23.40 ± 4.24	**3.97**	15.85 ± 2.74	2.80	14.98 ± 2.68	1.76	23.95 ± 3.94	**3.62**	31.60 ± 5.47	2.77	24.13 ± 3.97	**3.12**
Junior college	177.52 ± 25.87	(0.052)	37.17 ± 5.45	(0.054)	24.71 ± 3.84	**(0.019)**	16.57 ± 2.52	(0.062)	15.61 ± 2.88	(0.172)	25.02 ± 3.64	**(0.027)**	33.02 ± 4.91	(0.064)	25.43 ± 3.60	**(0.045)**
Undergraduate	172.68 ± 24.22		36.15 ± 5.06		23.89 ± 3.66	**b > a, c**	16.13 ± 2.34		15.19 ± 2.82		24.22 ± 3.45	**b > c**	32.16 ± 4.62		24.94 ± 3.44	**b > a**
Nursing is a first‐choice major for the college/school entrance examination																
Yes	178.46 ± 25.36	**3.91**	37.36 ± 5.37	**3.78**	24.82 ± 3.77	**3.82**	16.67 ± 2.46	**3.93**	15.68 ± 2.86	**2.89**	25.12 ± 3.58	**3.61**	33.21 ± 4.80	**3.97**	25.59 ± 3.53	**4.17**
No	170.18 ± 26.37	**(** * **)**	35.68 ± 5.48	**(** * **)**	23.62 ± 3.97	**(** * **)**	15.86 ± 2.59	**(** * **)**	15.00 ± 2.82	**(** * **)**	24.04 ± 3.71	**(** * **)**	31.62 ± 5.05	**(** * **)**	24.37 ± 3.69	**(** * **)**
I am willing to pursue a career in the nursing field in the future																
Yes	181.00 ± 25.31	**7.32**	37.91 ± 5.31	**7.29**	25.18 ± 3.79	**7.01**	16.89 ± 2.44	**6.93**	15.94 ± 2.88	**6.13**	25.48 ± 3.53	**7.13**	33.69 ± 4.81	**7.35**	25.90 ± 3.51	**6.90**
No	167.16 ± 24.42	**(** * **)**	35.00 ± 5.19	**(** * **)**	23.20 ± 3.63	**(** * **)**	15.61 ± 2.45	**(** * **)**	14.64 ± 2.64	**(** * **)**	23.58 ± 3.53	**(** * **)**	31.05 ± 4.64	**(** * **)**	24.07 ± 3.48	**(** * **)**

*Note:* The bolded t‐values in the table indicate significant differences.

* *p* < 0.001.
